# Reliable Disparity Estimation Using Multiocular Vision with Adjustable Baseline

**DOI:** 10.3390/s25010021

**Published:** 2024-12-24

**Authors:** Victor H. Diaz-Ramirez, Martin Gonzalez-Ruiz, Rigoberto Juarez-Salazar, Miguel Cazorla

**Affiliations:** 1 Instituto Politécnico Nacional—CITEDI, Ave. Instituto Politécnico Nacional 1310, Tijuana 22310, Mexico; mruiz@citedi.mx; 2 CONAHCYT, Instituto Politécnico Nacional—CITEDI, Ave. Instituto Politécnico Nacional 1310, Tijuana 22310, Mexico; rjuarezsa@conahcyt.mx; 3 Institute for Computer Research, University of Alicante, P.O. Box 99, 03080 Alicante, Spain; miguel.cazorla@ua.es

**Keywords:** adjustable baseline, disparity estimation, multiocular rectification, multiocular vision, stereo vision, three-dimensional reconstruction

## Abstract

Accurate estimation of three-dimensional (3D) information from captured images is essential in numerous computer vision applications. Although binocular stereo vision has been extensively investigated for this task, its reliability is conditioned by the baseline between cameras. A larger baseline improves the resolution of disparity estimation but increases the probability of matching errors. This research presents a reliable method for disparity estimation through progressive baseline increases in multiocular vision. First, a robust rectification method for multiocular images is introduced, satisfying epipolar constraints and minimizing induced distortion. This method can improve rectification error by 25% for binocular images and 80% for multiocular images compared to well-known existing methods. Next, a dense disparity map is estimated by stereo matching from the rectified images with the shortest baseline. Afterwards, the disparity map for the subsequent images with an extended baseline is estimated within a short optimized interval, minimizing the probability of matching errors and further error propagation. This process is iterated until the disparity map for the images with the longest baseline is obtained. The proposed method increases disparity estimation accuracy by 20% for multiocular images compared to a similar existing method. The proposed approach enables accurate scene characterization and spatial point computation from disparity maps with improved resolution. The effectiveness of the proposed method is verified through exhaustive evaluations using well-known multiocular image datasets and physical scenes, achieving superior performance over similar existing methods in terms of objective measures.

## 1. Introduction

Nowadays, many computer vision applications, such as robotics, autonomous navigation, augmented reality and medical imaging, require accurate estimation of 3D information from captured images. This 3D information enables computer vision systems to better understand and interact more efficiently and safely with the environment. Existing methods, such as structure from motion, time-of-flight cameras, and structured light projection, face challenges such as high computational cost, sensitivity to lighting variations, restricted field of view, and reduced performance in dynamic scenes.

Over the years, binocular stereo vision has been intensively investigated for 3D reconstruction, owing to its simple setup, cost-effectiveness, wide visual range, ease of data capture, and consistent results [1,2]. Conventionally, binocular vision-based 3D reconstruction involves four essential steps: image rectification to align the epipolar lines (the projection of the same 3D point onto the image planes of two cameras) parallel to the horizontal axis, detection of corresponding points in a 3D scene from stereo images, disparity estimation based on the location differences of the corresponding points, and retrieval of the 3D information through triangulation.

The accuracy of 3D reconstruction in binocular vision depends on the baseline separation between the cameras. A larger baseline improves disparity resolution by increasing the dynamic range of disparity for a given spatial point. This enables more accurate depth perception and the retrieval of detailed 3D information. However, it also increases the probability of disparity estimation errors due to large perspective differences in the captured stereo images. In contrast, a small baseline reduces the probability of disparity errors at the cost of lower depth resolution. Stereo vision with an adjustable baseline allows flexible capturing of scene images from different perspectives while providing accurate depth information. These features are highly valuable for tasks requiring depth perception and spatial understanding in dynamic scenes, for instance, in object detection, distance estimation, and obstacle avoidance in autonomous driving. Additionally, this approach can be useful for the construction of richer datasets for training and testing machine learning models.

In recent years, several approaches have been explored to improve the accuracy and robustness of stereo vision for a large baseline. One approach utilizes a grayscale binocular camera, a projector, and a color camera [3]. The projector displays a random pattern over the scene to add texture. The binocular and color cameras capture the textured scene, providing grayscale stereo images and a single color image. The disparity map is then computed by semi-global block matching from the stereo images [4], and the scene depth is obtained from the disparity map by triangulation. Finally, the 3D reconstruction is obtained by combining the depth map with the processed color image, where the projected pattern was suppressed using a long-pass filter. Although the added texture improves disparity estimation, this method is susceptible to perspective distortions due to a large baseline, increased calibration complexity, and greater sensitivity to lighting changes.

Another approach uses a trinocular stereo camera [5]. First, the disparity map between a reference camera and the central camera is estimated using the Census transform [6,7]. Next, disparity values between the reference and final cameras are estimated through a linear transformation. Each estimated disparity is scaled by the baseline ratio and adjusted with an additive value determined within a specified search interval. Although this approach improves robustness against perspective distortions by extending the baseline, the search interval for disparity estimation is not optimized. This can lead to unnecessary computations and disparity estimation errors. Additionally, this approach is limited to applications with a restricted field of view.

Another effective approach consists of using a multibaseline stereo camera [8,9,10,11]. A reliable technique within this approach [12] first estimates the disparity map between an image captured by a reference camera and those captured by the other cameras, using a combined weighted Census transformation [13] and sum of absolute differences (SAD-Census) [14]. Next, interpolation is applied to the computed disparity maps to recover the unknown disparity values between the reference image and the image with the largest baseline. Although this technique yields good results, it is computationally intensive. Additionally, the interpolation process can introduce artifacts, particularly in areas with significant disparity variation. Another effective technique uses a global-matching method to estimate the disparity map from stereo images with a long baseline [15]. First, image rectification aligns the captured image planes to be coplanar, exploiting the geometric structure of the multiocular array. Then, the disparity map for the longest baseline is incrementally constructed from the disparity maps of stereo images with shorter baselines, searching within a narrowed range deduced from numerical quantization errors. Although this technique is more tolerant to perspective differences and suitable for wide-field-of-view applications, its performance strongly depends on rectification. As the rectification method relies on assumptions about the geometry of the multiocular array, its performance can be degraded in the presence of scene perturbations. Additionally, the search range adaptation based on numerical quantization errors is not optimal, potentially leading to unnecessary computations and disparity errors.

Sophisticated learning techniques, like neural networks and deep artificial intelligence algorithms, have been explored for disparity estimation in stereo vision systems [16,17,18,19,20]. Despite showing promising results, these techniques still present important drawbacks, including the need for extensive labeled datasets, intensive computational requirements for training, and limited interpretability of resultant models.

The proposed approach integrates advanced computational and analytical optimization strategies to achieve robust performance in multiocular image rectification and disparity estimation, without relying on machine learning algorithms.

Table 1 summarizes representative existing techniques for stereo vision. It is worth noting that a widely explored approach relies on multiocular vision by gradually increasing the baseline. However, although this approach is effective, several limitations still need to be addressed, for instance, propagation of estimation errors while increasing the baseline, quantization errors due to image rectification, and inefficient computations caused by arbitrary search range specification for different baselines.

This work proposes an efficient method for reliable disparity estimation using multiocular vision with an adjustable baseline. By capturing several stereo images with a progressive baseline increase, the perspective differences among captured images change gradually. This allows us to better address undesired perspective distortions and achieve improved resolution in disparity estimation. First, a multiocular rectification method is formulated as a search problem using particle swarm optimization (PSO) [23], aimed to minimize both the rectification error and induced distortion. Next, a dense disparity map from the rectified images with the shortest baseline is computed using adaptive morphological correlation (AMC) [24]. Afterwards, the disparity map for the pair of rectified images with the subsequent larger baseline is efficiently computed within an optimized search range. This range is optimized by establishing a confidence interval that considers a disparity prediction and the expected dispersion of disparity. This process iterates until the disparity map for the stereo images with the longest baseline is obtained. The proposed approach improves the reliability of disparity estimation due to two main features. First, the proposed rectification method effectively minimizes quantization errors caused by incorrect epipolar alignment and distortion effects. Second, the adaptive optimization of the search interval minimizes matching errors, preventing excessive disparity error propagation and unnecessary computations. The main contributions of this research are described as follows:

- We developed a novel PSO-based method for multiocular image rectification that minimizes an objective function which balances epipolar constraints (horizontal aligment of epipolar lines) and induced distortion from detected point correspondences. This method is scalable from two to $N_{c}$ cameras and offers improved robustness over conventional approaches, as the population-based PSO optimization can yield good solution even in challenging situations.
- We present a reliable method for dense disparity estimation that iteratively improves disparity maps. Beginning with disparity estimation from short-baseline stereo images, the baseline is progressively extended to obtain disparity maps for images with the longest baseline. An adaptive search interval optimization is introduced to avoid unnecessary computations and minimize disparity estimation errors.
- We present a framework for three-dimensional imaging based on multiocular vision, enabling accurate characterization of physical scenes and reliable spatial point computation from a single multiocular image capture.

The paper is organized as follows. Section 2 introduces the essential concepts of multiocular vision geometry. Section 3 presents details of 3D imaging using multiocular vision, employing the proposed approach for reliable disparity estimation with an adjustable baseline. A detailed explanation of the suggested rectification method for multiocular images is provided. Additionally, the proposed iterative approach for disparity estimation using multiocular images with adjustable baseline is discussed. Section 4 presents the results of the proposed method for reliable disparity estimation. First, the performance of the introduced rectification method is evaluated in terms of objective measures, using images from well-known datasets and multiocular images captured on an experimental platform. Next, the performance of the proposed disparity estimation approach for multiocular images is evaluated and discussed in terms of objective criteria. Finally, 3D reconstruction results using the proposed method are presented and discussed. Section 5 summarizes the conclusions.

## 2. Multiocular Vision Geometry

**Figure 1:**
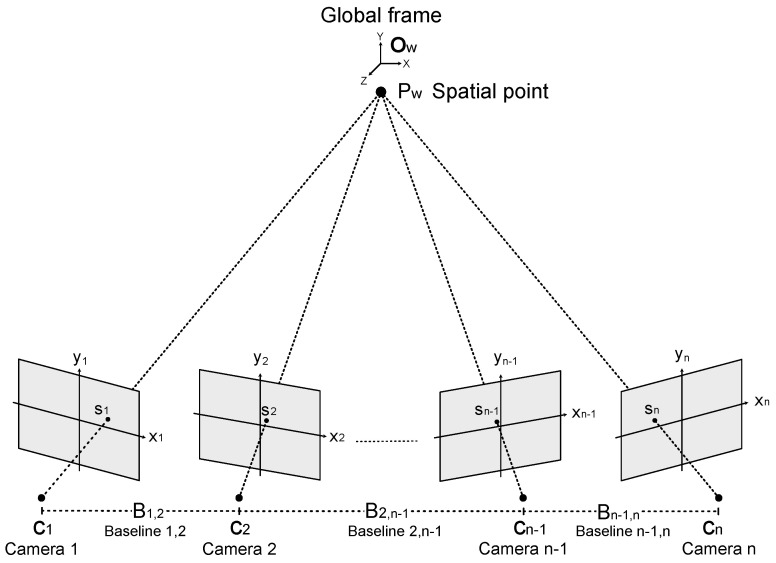
Optical setup of a multiocular vision system.

Consider the multiocular camera setup depicted in Figure 1. This setup is composed of $N_{c}\geq 2$ cameras $\left\{c_{i}|i=1,\ldots ,N_{c}\right\}$, with separation distance (baseline) between cameras *i* and *j* given by $B_{i,j}$. The camera $c_{i}$ images a spatial point $P_{w}={\left[X,Y,Z\right]}^{T}$ defined in a global frame $O_{w}$ as the point

(1) $$s_{i}=H_{1}^{-1}\left[C_{i}H_{1}\left[P_{w}\right]\right],$$

where $H_{1}\left[p\right]={\left[p,1\right]}^{T}$ and $H_{1}^{-1}\left[H_{1}\left[p\right]\right]=p$, are the homogeneous and inverse homogeneous operators [25], respectively, and $C_{i}$ is the 3×4 projection matrix of camera $c_{i}$ which maps from 3D space to the 2D camera image plane.

The camera matrix is given by $C_{i}=K_{i}M_{i}$, where $K_{i}$ and $M_{i}$ are intrinsic and extrinsic parameters matrices, respectively. The intrinsic parameter matrix can be defined as [26]

(2) $$K_{i}=\left[\begin{array}{ccc} f_{i}m_{x_{i}} & \gamma_{i} & x_{0_{i}}\\ 0 & f_{i}m_{y_{i}} & y_{0_{i}}\\ 0 & 0 & 1 \end{array}\right],$$

where $f_{i}$ is the focal length, $“m_{x_{i}},m_{y_{i}}”$ specify the pixel size, $“x_{0_{i}},y_{0_{i}}”$ are the coordinates of the principal point, and $\gamma_{i}$ is the skew parameter. Additionally, the 3×4 extrinsic parameter matrix is given by [27]

(3) $$M_{i}=\left[R_{i}^{T},-R_{i}^{T}t_{i}\right],$$

where $R_{i}$ is a 3×3 rotation matrix and $t_{i}$ is the 3×1 translation vector, both with respect to the global frame $O_{w}$.

According to the epipolar geometry, the imaged points by cameras $c_{i}$ and $c_{j}$ satisfy the following condition [28]:

(4) $$H_{1}^{T}\left[s_{j}\right]F_{i,j}H_{1}\left[s_{i}\right]=0;\;\mathrm{for}\;i<j\leq n,$$

where $F_{i,j}$ is the 3×3 fundamental matrix that describes the projective geometry between $c_{i}$ and $c_{j}$. The matrix $F_{i,j}$ depends solely on the intrinsic parameters and relative pose of $c_{i}$ and $c_{j}$. This matrix can be obtained from the camera parameters as follows [26]:

(5) $$F_{i,j}=H_{1}\left[e_{j}\right]\times K_{j}M_{j}{\left[M_{i}^{T}\left(K_{i}^{T}K_{i}\right)M_{i}\right]}^{-1}K_{i}^{T}M_{i}^{T},$$

where $e_{j}=H_{1}^{-1}\left[C_{j}R_{j}^{T}\left(t_{i}-t_{j}\right)\right]$ is the epipole in the image plane of $c_{j}$, and “×” denotes the cross-product operator.

A particularly useful case is when the cameras $c_{i}$ and $c_{j}$ are perfectly aligned horizontally. Without loss of generality, consider that the location of camera $c_{i}$ coincides with the origin of the global frame $O_{w}$. Thus, the extrinsic parameters are

(6) $$M_{i}=\left[I_{3},0_{3}\right],\;\mathrm{and}\;\;M_{j}=\left[I_{3},b_{i,j}\right],$$

where $I_{3}$ is the 3×3 identity matrix, $0_{3}$ is the 3×1 zero vector, and $b_{i,j}={\left[B_{i,j},0,0\right]}^{T}$ is a translation vector specified by the baseline $B_{i,j}$ between cameras $c_{i}$ and $c_{j}$. Consequently, the epipole $e_{j}$ is relocated at infinity as

(7) $$H_{0}\left[e_{j}\right]={\left[1,0,0\right]}^{T},$$

and the fundamental matrix is

(8) $${\tilde{F}}_{i,j}=H_{0}\left[e_{j}\right]\times K_{i}K_{j}^{-1}.$$

For cameras with the same intrinsic parameters $\left(K_{i}=K_{j}\right)$, the canonical fundamental matrix is

(9) $$\tilde{F}=\left[\begin{array}{ccc} 0 & 0 & 0\\ 0 & 0 & -1\\ 0 & 1 & 0 \end{array}\right].$$

## 3. Three-Dimensional Imaging Using Multiocular Vision

Let $\left\{P_{k}={\left[X_{k},Y_{k},Z_{k}\right]}^{T}|k=1,\ldots ,N_{k}\right\}$ be a set of points of an object specified in the global frame $O_{w}$. These points are imaged by the cameras $\left\{c_{i}|i=1,\ldots ,N_{c}\right\}$ as shown in Figure 1, resulting in the points $\left\{s_{k,i}|k=1,\ldots ,N_{k}\right\}$ as given in Equation (1). It should be noted that if Equation (6) is satisfied, the point $s_{k,j}$ can be specified in terms of the point $s_{k,i}$ as follows:

(10) $$s_{k,j}=s_{k,i}+H_{0}\left[\tau_{i,j}\left(k\right)\right];\;\mathrm{for}\;i<j\leq N_{c},$$

where $\tau_{i,j}\left(k\right)$ is the disparity between the points $\left(s_{k,i}↔s_{k,j}\right)$. Therefore, a solution vector ${\left[H_{1}\left[P_{k}\right],\lambda_{0},\lambda_{1}\right]}^{T}$ can be obtained from Equations (1) and (10) by solving

(11) $$\left[\begin{array}{ccc} K_{i}M_{i} & H_{1}\left[s_{k,i}\right] & 0_{3}\\ K_{j}M_{j} & 0_{3} & H_{1}\left[s_{k,i}+H_{0}\left[\tau_{i,j}\left(k\right)\right]\right] \end{array}\right]\left[\begin{array}{c} H_{1}\left[P_{k}\right]\\ \lambda_{0}\\ \lambda_{1} \end{array}\right]=0_{6},$$

where $“\lambda_{0}$, $\lambda_{1}”$ are arbitrary scalar values and $0_{6}$ is the 6×1 zero vector. The spatial coordinates of $P_{k}$ can be obtained from the corresponding points $\left(s_{k,i}↔s_{k,j}\right)$ as

(12) $${\left[X_{k},Y_{k},Z_{k}\right]}^{T}=H_{i}^{-1}\left[H_{1}\left[P_{k}\right]\right].$$

It is worth mentioning that to solve Equation (11) and compute the spatial coordinates from Equation (12), it is necessary to know the camera parameters $“K_{i},K_{j}”$ and $“M_{i},M_{j}”$, as well as the disparity values $\tau_{i,j}\left(k\right)$ for all corresponding points $\left(s_{k,i}↔s_{k,j}\right)$ in the captured images $S_{i}\left(x,y\right)$ and $S_{j}\left(x,y\right)$.

The intrinsic parameters $\left\{K_{i}|i=1,\ldots ,N_{c}\right\}$ can be obtained by calibration [26]. However, the disparity values $\tau_{i,j}\left(k\right)$ need to be estimated by matching the points $s_{k,i}$ in the image $S_{i}\left(x,y\right)$ with the corresponding points $s_{k,j}$ in the image $S_{j}\left(x,y\right)$, scanning along the epipolar lines. This task is simplified if $S_{i}\left(x,y\right)$ and $S_{j}\left(x,y\right)$ are previously rectified, satisfying Equations (6) and (7).

In Section 3.1, we introduce the proposed rectification method for multiocular images that satisfy the epipolar conditions in Equations (6) and (7) while minimizing projective distortion in the resultant rectified images. Additionally, in Section 3.2 we present the proposed method for reliable disparity estimation from a set of captured multiocular images.

### 3.1. Multiocular Image Rectification

In essence, the rectification process applies a projective transformation to each image captured by a multiocular setup, as shown in Figure 1. This transformation aims to relocate the epipoles of the transformed images to infinity, as in Equation (7), while minimizing the resultant projective distortion.

A projective transformation of the image points $s_{k,i}$ can be carried out as

(13) $${\tilde{s}}_{k,i}=H_{1}^{-1}\left[G_{i}H_{1}\left[s_{k,i}\right]\right],$$

where $G_{i}$ is a 3×3 homography matrix given as [1]

(14) $$G_{i}=K_{i}\left[r_{1i},r_{2i},-R_{i}^{T}t_{i}\right],$$

with $“r_{1i}$, $r_{2i}”$ representing the row vectors of the rotation matrix $R_{i}^{T}=\left[r_{1i},r_{2i},r_{3i}\right]$. Note that $G_{i}$ can be constructed from the camera parameters $K_{i}$ and $M_{i}$. However, since $K_{i}$ can be previously obtained by calibration, $G_{i}$ can be parameterized by six extrinsic parameters, specifically three translation parameters $t_{i}={\left[t_{x,i},t_{y,i},t_{z,i}\right]}^{T}$ and three rotation parameters $\rho_{i}={\left[\alpha_{i},\psi_{i},\phi_{i}\right]}^{T}$ characterizing the rotation matrix as

(15) $$R\left(\rho_{i}\right)=R_{z}\left(\phi_{i}\right)R_{y}\left(\psi_{i}\right)R_{z}\left(\alpha_{i}\right),$$

where the Euler sequence “z→y→z” is considered.

Multiocular image rectification using the setup depicted in Figure 1 requires the construction of $N_{c}$ rectifying homographies. This task can be formulated as an optimization problem of $6N_{c}$ parameters. Note that this problem is multi-dimensional and non-convex. Therefore, the use of a population-based optimization method, such as PSO, is particularly appropriate. Consider a set $\left\{\left(m_{k,i}↔m_{k,j}\right)|k=1,\ldots ,N_{m}\right\}$ of detected corresponding points in the input images $S_{i}\left(x,y\right)$ and $S_{j}\left(x,y\right)$, respectively. Furthermore, let

(16) $$v={\left[\left(t_{1}^{T},\rho_{1}^{T}\right),\left(t_{2}^{T},\rho_{2}^{T}\right),\ldots ,\left(t_{N_{c}}^{T},\rho_{N_{c}}^{T}\right)\right]}^{T},$$

be a $6N_{c}\times 1$ parameter vector that allows the construction of $N_{c}$ rectifying homographies $\left\{G_{i}|i=1,\ldots ,N_{c}\right\}$ as given in Equation (14). The vector *v* is defined in the parameter space $\Omega_{V}\in R^{6N_{c}}$, with geometrical bounds imposed by the extrinsic parameters of position $t_{i}$ and orientation $\rho_{i}$. Thus, a given parameter vector *v* encodes a solution for the rectifying homographies $\left\{G_{i}|i=1,\ldots ,N_{c}\right\}$. Additionally, an evaluation function $J\left(v\right)=eval\left(v,v^{*}\right)$ is required to quantify the distance of a potential solution *v* with respect to the optimal solution

(17) $$v^{*}=\underset{v}{\arg\min}\left\{J(v)\right\}.$$

The proposed rectification method optimizes two objective criteria: the rectification error $J_{e}\left(v\right)$ and the projective distortion $J_{d}\left(v\right)$. The $J_{e}\left(v\right)$ criterion measures the accuracy of satisfying the epipolar constraints given in Equation (4) when Equations (6) and (7) are met, and is given as

(18) $$J_{e}\left(v\right)=\sum_{j=2}^{N_{c}}\sum_{k=1}^{N_{m}}H_{1}{\left[m_{k,1}\right]}^{T}G_{1}^{T}\left(v\right)\tilde{F}G_{j}\left(v\right)H_{1}\left[m_{k,j}\right],$$

where $\tilde{F}$ is the canonical fundamental matrix in Equation (9). Note that a vector *v* which minimizes Equation (18) satisfies the epipolar constraints for rectified images given in Equation (4). However, an undesirable projective distortion will be induced to the resultant rectified images. The null distortion only occurs when the rectifying homographies are $G_{i}\left(v\right)=I_{3}$. Thus, to quantify the induced distortion, consider that a reference rectified image with null distortion has corner points $q={\left[q_{x},q_{y}\right]}^{T}$, where

(19) $$\begin{array}{cc} q_{x} & ={\left[-1,-1,1,1\right]}^{T},\\ q_{y} & ={\left[1,-1,-1,1\right]}^{T}. \end{array}$$

Hence, the center point in homogeneous coordinates is

(20) $$q_{0}={\left[0,0,1\right]}^{T}.$$

Note that the lines

(21) $$\begin{array}{cc} l_{1} & =H_{1}\left[{\left[q_{x}\left(1\right),q_{y}\left(1\right)\right]}^{T}\right]\times H_{1}\left[{\left[q_{x}\left(4\right),q_{y}\left(4\right)\right]}^{T}\right],\\ l_{2} & =H_{1}\left[{\left[q_{x}\left(2\right),q_{y}\left(2\right)\right]}^{T}\right]\times H_{1}\left[{\left[q_{x}\left(3\right),q_{y}\left(3\right)\right]}^{T}\right], \end{array}$$

intersect the upper and bottom corner points, respectively, and are parallel. The projective transformations of $q_{0}$, $l_{1}$, and $l_{2}$ using the homography $G_{i}\left(v\right)$ are given as [29]

(22) $${\tilde{q}}_{0}\left(v\right)=G_{i}\left(v\right)q_{0},$$

(23) $${\tilde{l}}_{1}\left(v\right)={\left(G_{i}^{-1}\left(v\right)\right)}^{T}l_{1},$$

and

(24) $${\tilde{l}}_{2}\left(v\right)={\left(G_{i}^{-1}\left(v\right)\right)}^{T}l_{2}.$$

Thus, the projective distortion induced by the parameter vector *v* can be quantified as

(25) $$J_{d}\left(v\right)=\sum_{i=1}^{N_{c}}{∥q_{0}-{\tilde{q}}_{0}\left(v\right)∥}_{2}+g_{3,i}^{T}\left(v\right)\left[l_{1}+l_{2}\right],$$

where $g_{3,i}\left(v\right)$ is the third column of the inverse homography $G_{i}^{-1}\left(v\right)=\left[g_{1,i}\left(v\right),g_{2,i}\left(v\right),g_{3,i}\left(v\right)\right]$. It should be noted that for the case of null distortion, it is obtained ${\tilde{q}}_{0}\left(v\right)=q_{0}$, ${\tilde{l}}_{1}\left(v\right)=l_{1}$, ${\tilde{l}}_{2}\left(v\right)=l_{2}$, and $J_{d}\left(v\right)=0$.

**Figure 2:**
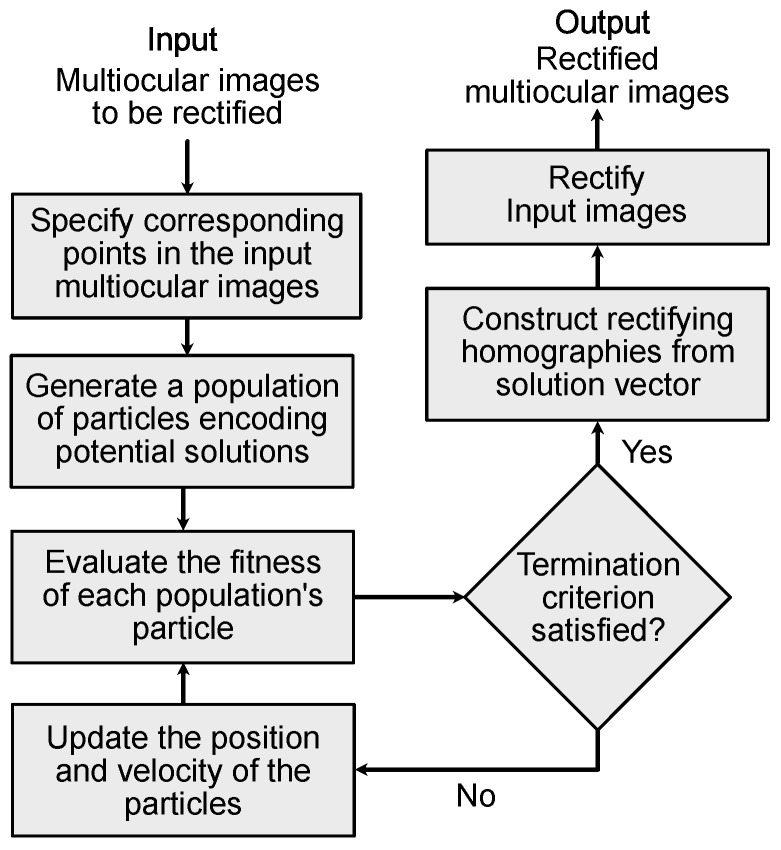
Block diagram of the proposed PSO-based method for multiocular image rectification.

The proposed rectification method searches for an optimal parameter vector $v^{*}$ with the form of Equation (16), using PSO (Appendix A). This is achieved by minimizing both the rectification error $J_{e}\left(v\right)$ and the projective distortion $J_{d}\left(v\right)$, given in Equations (18) and (25), respectively. The block diagram of the proposed rectification method is shown in Figure 2, with the steps (S1, …, S5) detailed below.

S1 Detect a set of corresponding points $M=\{\left(m_{k,1}↔m_{k,j}\right)|j=2,\ldots ,N_{c};\;k=1,\ldots ,N_{k}\}$, in the input images $\left\{S_{i}\left(x,y\right)|i=1,\ldots ,N_{c}\right\}$. The index *j* identifies the camera that captured the images, and *k* is the index of the corresponding point.
S2 Construct a particle swarm $V=\{v_{i}|i=1,\ldots ,N_{v}\}$, where each particle $v_{i}$ represents of a unique parameter combination as given in Equation (16). Initially, particles are randomly generated using a uniform distribution within predefined geometric bounds for the extrinsic parameters.
S3 Evaluate the fitness of each particle of the swarm as follows. For a given particle $v_{i}$, construct the homographies $\left\{G_{i}|i=1,\ldots ,N_{c}\right\}$ using Equation (14). Next, perform a projective transformation of the corresponding points in *M* with each homography $G_{i}\left(v\right)$ and compute the rectification error using Equation (18). Afterwards, perform a projective transformation of the points *q* and $q_{0}$ with the homographies $G_{i}\left(v\right)$, as given in Equations (22)–(24). Then, compute $J_{d}\left(v\right)$ with Equation (25). Finally, evaluate the fitness as a trade-off between $J_{e}\left(v\right)$ and $J_{d}\left(v\right)$ as
  (26) $$J\left(v_{i}\right)=\eta J_{e}\left(v_{i}\right)+\left(1-\eta \right)J_{d}\left(v_{i}\right),$$
  where η∈(0,1) is a scalar coefficient given by the user.
S4 Verify if a particle $v_{i}$ underpasses a prespecified fitness value or if a maximum number of iterations is reached. If *true*, set the current solution vector as $v^{*}$ and proceed to next step. Otherwise, update the position and velocity of the particles of the swarm by applying the search operators given in Equation (A1), and then proceed to Section 3.1.
S5 Construct the homographies $\left\{G_{i}|i=1,\ldots ,N_{c}\right\}$ from $v^{*}$ and rectify the input multiocular images by performing the projective transformation in Equation (13).

### 3.2. Disparity Estimation from Multiocular Images

The disparity values $\tau_{i,j}\left(k\right)$ between the imaged points $\left(s_{k,i}↔s_{k,j}\right)$, as given in Equation (10), are required to compute the coordinates of spatial points using Equations (11) and (12). In rectified multiocular images, disparity occurs only along the *x* axis. However, for a given point $s_{k,i}$, the location of the corresponding point $s_{k,j}$ is unknown and needs to be determined.

Let $w_{k,i}$ be an W×1 vector containing the intensity values of imaged points in the close vicinity of $s_{k,i}$ in image $S_{i}\left(x,y\right)$. Additionally, let $w_{k,j}^{\tau_{x}}$ be an W×1 vector with the intensity values of the imaged points in the vicinity of $s_{k,i}$ in image $S_{j}\left(x,y\right)$, displaced by $\tau_{x}$ along the *x* axis. In this case, disparity estimation can be formulated as follows:

(27) $$\tau_{i,j}\left(k\right)=\underset{\tau_{x}\in \Delta_{\tau }}{\arg\min}\left\{diss\left(w_{k,i},w_{k,j}^{\tau_{x}}\right)\right\},$$

where diss(a,b) quantifies the dissimilarity of *a* and *b*, and $\Delta_{\tau }$ is a search interval given as

(28) $$\Delta_{\tau }=\left[\tau_{\min},\tau_{\min+1},\ldots ,\tau_{\max}\right].$$

Over the years, several effective matching methods have been proposed to estimate the disparity from stereo images, as given in Equation (27) [2,30]. In this work, we utilize the AMC-based approach [24] which minimizes the Binary-Dissimilarity-to-Matching Ratio (BDMR). In the multiocular setup shown in Figure 1, the disparity $\tau_{1,N_{c}}\left(k\right)$ between $c_{1}$ and $c_{N_{c}}$ enables more detailed reconstructions using Equation (12) considering the disparity values $\tau_{1,2}\left(k\right)$ between $c_{1}$ and $c_{2}$. This is because the baseline $B_{1,N_{c}}$ is larger than $B_{1,2}$. However, computing $\tau_{1,N_{c}}\left(k\right)$ is more challenging than computing $\tau_{1,2}\left(k\right)$ due to greater uncertainties in the captured images caused by perspective variations and partial occlusions.

To compute $\tau_{1,2}\left(k\right)$ using Equation (27), the search interval $\Delta_{\tau_{1,2}}$ for each point is defined by Equation (28). However, for the camera pair “$c_{1},c_{N_{c}}$” the search interval $\Delta_{\tau_{1,N_{c}}}$ for $\tau_{1,N_{c}}\left(k\right)$ increases significantly compared to $\Delta_{\tau_{1,2}}$ as [8]

(29) $$\Delta_{\tau_{1,N_{c}}}=\left(\frac{B_{1,N_{c}}}{B_{1,2}}\right)\Delta_{\tau_{1,2}}.$$

Thus, a direct computation of $\tau_{1,N_{c}}\left(k\right)$ along $\Delta_{\tau_{1,N_{c}}}$ is unfeasible due to the high probability of estimation errors caused by significant perspective distortions and larger search space.

Generally speaking, the disparity of a *k*-th point imaged by the camera pair $“c_{1},c_{1+i}”$ is $\tau_{1,1+i}\left(k\right)$. The baseline $B_{1,1+i}$ of this camera pair is shortest when i=1. If $\tau_{1,1+i}\left(k\right)$ is known, the disparity for the camera pair with a larger baseline, i.e., when i=2, can be predicted as

(30) $${\tilde{\tau }}_{1,i+1}\left(k\right)=\frac{B_{1,i+1}}{B_{1,i}}\tau_{1,i}\left(k\right).$$

This prediction is validated or corrected using Equation (27) by searching within a reduced interval. It is necessary to consider that the disparity predictions, as in Equation (30), are associated with the number of cameras in the multiocular imaging system. Other prediction techniques, such as the Kalman filter [31], could also be considered. However, this filter is designed to estimate the state of a linear dynamic system from noisy measurements and typically requires multiple measurements to converge. In a multiocular imaging system as shown in Figure 1, the number of cameras is generally within a practical range.

Instead, consider that a disparity prediction $\tilde{\tau }$, as in Equation (30), is a random variable with Gaussian distribution denoted as $\tilde{\tau }∼N\left(\mu_{\tilde{\tau }},\sigma_{\tilde{\tau }}^{2}\right)$, where $\mu_{\tilde{\tau }}$ is the expected value and $\sigma_{\tilde{\tau }}^{2}$ is the variance. Note that the predicted disparity values using Equation (30) for all imaged scene points, can be considered a large set of independent and identically distributed random variables. Therefore, by the Central Limit Theorem, these values can be approximated by a Gaussian distribution. The probability of $\tilde{\tau }$ to be located within the arbitrary interval [−α,α] is

(31) $$P\left(-\alpha <\tilde{\tau }<\alpha \right)=\frac{1}{\sqrt{2\pi \sigma_{\tilde{\tau }}^{2}}}\int_{-\alpha }^{\alpha }\exp\left[\frac{{\left(\tilde{\tau }-\mu_{\tilde{\tau }}\right)}^{2}}{2\sigma_{\tilde{\tau }}^{2}}\right].$$

Note that by performing the transformation $\eta =\left(\tilde{\tau }-\mu_{\tilde{\tau }}\right)/\sigma_{\tilde{\tau }}$, the resulting random variable is η∼N(0,1), and

(32) $$P\left(-\alpha <\eta <\alpha \right)=P\left(-\alpha <\frac{\tilde{\tau }-\mu_{\tilde{\tau }}}{\sigma_{\tilde{\tau }}}<\alpha \right).$$

Therefore, from Equation (32) the expected disparity value $\mu_{\tilde{\tau }}$ is located within the interval $\mu_{\tilde{\tau }}\in \left[\tilde{\tau }\pm \alpha \sigma_{\tilde{\tau }}\right]$. Hence, $\tilde{\tau }$ is a disparity prediction which can be obtained by Equation (30). Moreover, $\sigma_{\tilde{\tau }}$ denotes the dispersion of the predicted disparity $\tilde{\tau }$. According to the triangulation principle, far imaged points produce shorter disparities, whereas near imaged points produce larger disparity values.

Consequently, for a predicted disparity ${\tilde{\tau }}_{1,i+1}>\tau_{min}$ as given in Equation (30), an estimate of $\sigma_{\tilde{\tau }}$ is

(33) $$\sigma_{\tilde{\tau }}=\sqrt{\frac{1}{{\tilde{\tau }}_{1,i+1}-1}\sum_{j=1}^{{\tilde{\tau }}_{1,i+1}}{\left[\Delta_{\tau }\left(j\right)-\mu_{\Delta_{\tau }}\right]}^{2}},$$

where $\mu_{\Delta_{\tau }}=\left(\tau_{min}+{\tilde{\tau }}_{1,i+1}\right)/2$. Therefore, from Equations (32) and (33) the optimized search interval for a predicted disparity ${\tilde{\tau }}_{1,i+1}$, is

(34) $$\Delta_{\tilde{\tau }}=\left[{\tilde{\tau }}_{1,i+1}-\epsilon_{{\tilde{\tau }}_{min}},\ldots ,{\tilde{\tau }}_{1,i+1}+\epsilon_{{\tilde{\tau }}_{max}}\right],$$

where $\epsilon_{{\tilde{\tau }}_{min}}=\max\left\{\tau_{min},\alpha \sigma_{\tilde{\tau }}\right\}$ and $\epsilon_{{\tilde{\tau }}_{max}}=\min\left\{\alpha \sigma_{\tilde{\tau }},\tau_{max}\right\}$. According to Equation (34), the parameter α specifies the confidence that the value of the random variable $\tilde{\tau }$ is located within the specified interval with a specific probability.

**Figure 3:**
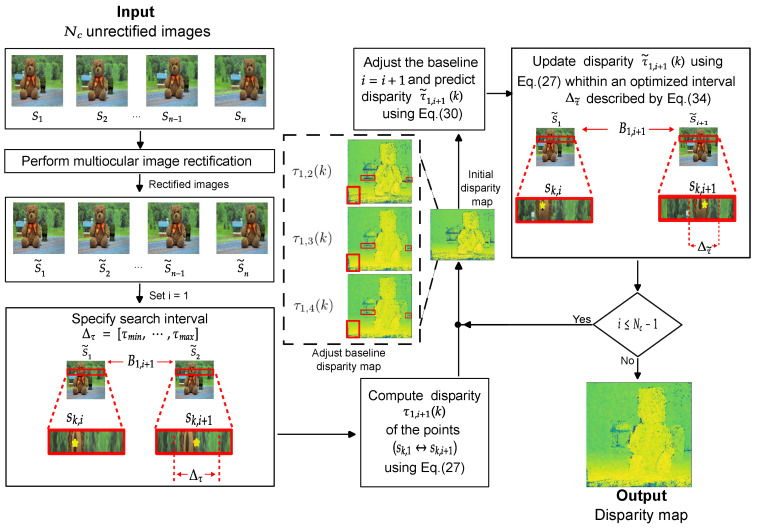
Diagram of the proposed method for disparity estimation with an adjustable baseline.

The diagram of the proposed disparity estimation method is shown in Figure 3, with the steps described below.

S1 Capture a set of $N_{c}>2$ images as depicted in the setup shown in Figure 1.
S2 Rectify the captured images using the proposed PSO-based method described in Section 3.1.
S3 Set i=1 and specify the search interval $\Delta_{\tau }$ as given in Equation (28).
S4 Compute the disparity $\tau_{1,i+1}\left(k\right)$ of all corresponding points $\left(s_{k,1}↔s_{k,i+1}\right)$ in the rectified images ${\tilde{S}}_{1}\left(x,y\right)$ and ${\tilde{S}}_{i+1}\left(x,y\right)$ as described in Equation (27).
S5 Adjust the baseline by setting i=i+1 and predict the disparity values ${\tilde{\tau }}_{1,i+1}\left(k\right)$ using Equation (30).
S6 Update the disparity estimate $\tau_{1,i+1}\left(k\right)$ using Equation (27) within an optimized search interval $\Delta_{\tilde{\tau }}$ obtained with Equation (34).
S7 Verify if $i\leq (N_{c}-1)$. If *true*, proceed to S5. Otherwise, $\left\{\tau_{1,i+1}\left(k\right)|k=1,\ldots ,N_{k}\right\}$ are the final disparity estimates.

## 4. Results

This section presents and discusses the results of the proposed rectification and disparity estimation methods for multiocular vision with an adjustable baseline. In the first experiment, we evaluate the performance of the proposed rectification method using stereo images from two well-known datasets: the Media Communication Lab Real Stereo (MCL-RS) [32] from the University of Southern California and the INRIA Syntim database [33]. The rectification performance is objectively quantified in terms of the rectification error and perspective distortion. The performance of the proposed method is compared with that of three existing rectification methods. Additionally, the rectification performance is evaluated using real images captured on a constructed experimental platform. In the second experiment, the performance of the proposed disparity estimation method is evaluated using multiocular images with a variable baseline from the Middlebury stereo dataset [34,35,36]. The results are discussed and compared with those obtained with an existing multi-baseline stereo matching method. Finally, the third experiment validates the practical feasibility of the proposed approach by presenting 3D reconstructions of several real scenes from multiocular images captured on the constructed experimental platform.

### 4.1. Rectification Performance Evaluation

The performance of the proposed rectification method is tested using 40 stereo images, combined from the MCL-RS [32] and Syntim [33] datasets. The MCL-RS dataset consists of five indoor and fifteen outdoor scenes with varying textures, contrast conditions, non-homogeneous illumination, and differences in zoom and camera poses. The Syntym dataset includes 21 indoor scenes, two outdoor scenes, and seven synthetic scenes, with variations in texture, blurring, and illumination. In this experiment, $N_{k}=100$ corresponding points are detected in the input images $\left\{S_{i}\left(x,y\right)|i=1,\ldots ,N_{c}\right\}$ using the scale-invariant feature transform (SIFT) algorithm [37]. The detected points are further analyzed utilizing the RANSAC algorithm [38] for outlier rejection. Next, the rectifying homographies $\left\{G_{i}|i=1,\ldots ,N_{c}\right\}$ are computed using the proposed rectification algorithm shown in Figure 2, with the following parameterization: swarm size $N_{v}=500$, η=0.95, $c_{1}=0.5$, $c_{2}=0.3$, and ω=0.9. Note that the cognitive ($c_{1}$) and social ($c_{2}$) acceleration coefficients are crucial in determining the accuracy and convergence of particles within the swarm. These coefficients balance the particles’ reliance on their own trajectories and the influence of the global swarm trajectory. In our experiments, setting $c_{1}=2.0$ and $C_{2}=2.5$ led to faster convergence but with increased rectification error. In contrast, setting $c_{1}=0.5$ and $c_{2}=0.3$ minimized the rectification error, though requiring more iterations. Finally, the input images are rectified using the resultant homographies $G_{i}$ using Equation (13). The rectification error is measured as the mean absolute difference among the rectified corresponding points $\left({\tilde{m}}_{k,1}↔{\tilde{m}}_{k,j}\right)$ [39] as

(35) $$e_{rec}=\frac{1}{N_{c}N_{m}}\sum_{j=2}^{N_{c}}\sum_{k=1}^{N_{m}}\left|{\tilde{m}}_{k,1}^{y}-{\tilde{m}}_{k,j}^{y}\right|,$$

where ${\tilde{m}}_{k,1}={\left[{\tilde{m}}_{k,1}^{x},{\tilde{m}}_{k,1}^{y}\right]}^{T}$ and ${\tilde{m}}_{k,j}={\left[{\tilde{m}}_{k,j}^{x},{\tilde{m}}_{k,j}^{y}\right]}^{T}$. Furthermore, the perspective distortion is quantified by the aspect ratio AR and orthogonality angle (θ) as suggested in [40]. The performance of the proposed rectification method is compared with that of the methods by Fusiello et al. [39], Xiao et al. [41] (direct self-rectification, DSR), and Juarez-Salazar et al. [27] (stereo pase rectification, SPR), using $N_{c}=2$ images (binocular stereo). It is worth mentioning that the proposed method employs PSO for optimization, whereas Fusiello et al. [39] uses Levenberg–Marquardt, DSR [41] applies RANSAC, and SPR [27] utilizes Gauss–Newton for optimization.

**Figure 4:**
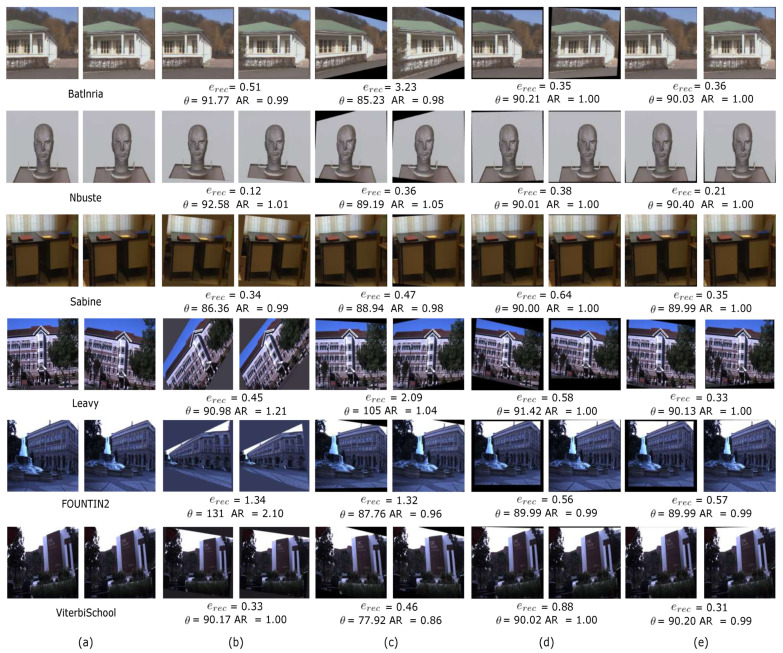
Stereo image rectification results. (**a**) Unrectified test images. Rectified images obtained using: (**b**) Fusiello et al. [42], (**c**) Juarez-Salazar et al. [27], (**d**) DSR [41], and (**e**) proposed method.

The proposed method was implemented in Python 3.12.2 with NumPy 1.24.4, OpenCV 4.9.0, Numba 0.59.1, and PySwarms 1.13.0 libraries, on a personal computer equipped with an Intel Core I7 1.7 GHz processor, 16 GB of RAM, and 64 bit Windows 11 operating system. Figure 4a shows several unrectified test images, whereas Figure 4b–e show the corresponding rectified images obtained with the proposed and considered existing methods. All tested methods successfully rectify the input images. However, the proposed method outperforms all tested methods, producing the lowest rectification error and minimal induced distortion. Table 2 presents the mean value and standard deviation of $e_{rec}$, AR, and θ measures for the rectification of 40 test scenes from the considered datasets. It should be noted that the proposed and DSR methods achieve the best performance. However, the proposed method outperforms the DSR method in all considered measures. The method by Fusiello yields very good results in terms of $e_{\mathrm{rec}}$. However, it induces a pronounced distortion in the rectified images in several cases. The SPR method produces the worst results. This is primarily due to its sensitivity to quantization errors in the detection of corresponding points. Based on these results, it can be seen that the use of a population-based optimization method, such as PSO, improves the robustness of the rectification process by avoiding suboptimal solutions through effectively exploring the solution space. This allows the discovery of better solutions even in scenarios with complex geometries compared to conventional optimization methods such as Gauss–Newton, Levenberg–Marquardt, or RANSAC.

**Figure 5:**
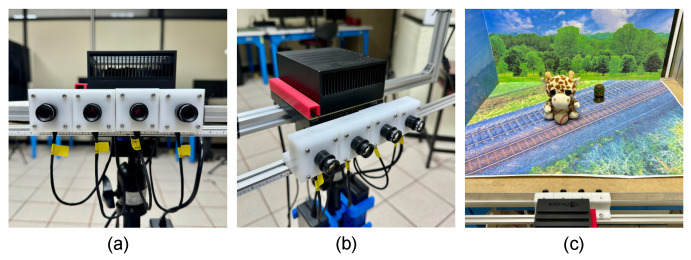
Constructed laboratory platform for experiments. (**a**) Frontal view of the multiocular camera. (**b**) Side view of the multiocular camera. (**c**) Test scene captured by the experimental multiocular platform.

**Figure 6:**
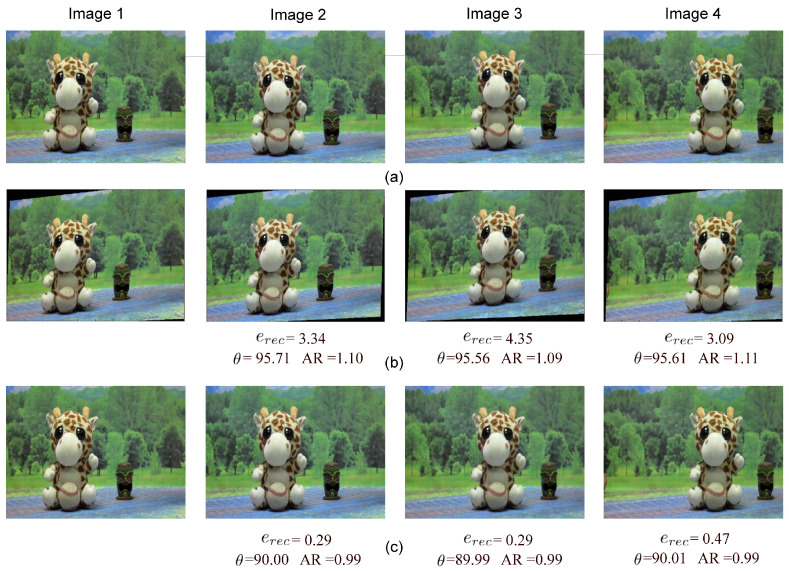
Multiocular image rectification results from a real scene captured with the experimental platform shown in Figure 5. (**a**) Unrectified input images. Rectified images obtained using: (**b**) Yang et al. [44] method and (**c**) proposed method.

The proposed rectification method was also tested for multiocular image rectification using 25 real scenes captured with the experimental platform shown in Figure 5. This platform consists of a multiocular array of four 13 MP 4-Lane MIPI CSI-2 e-CAM130A cameras, each with a 7 mm focal length lens. The cameras are aligned horizontally with a 45 mm baseline between each camera, as illustrated in Figure 5a,b. The cameras are controlled through a 64 GB NVIDIA Jetson Xavier AGX development board. All captured images are RGB color with a resolution of 1920 × 1080 pixels. The intrinsic parameters of the cameras were obtained using three widely known calibration methods: the Direct Linear Transformation [1], the distorted pinhole method [43], and the method proposed by Zhang et al. [26]. The performance of the proposed method is compared with that of a similar method for multiocular image rectification by Yang et al. [44]. Figure 6a shows test images captured of a scene using the experimental platform depicted in Figure 5c. Additionally, Figure 6b,c present the resultant rectified images obtained using the method by Yang and the proposed method, respectively. It can be seen that the proposed method yields significantly better results than Yang’s method. Table 3 presents the mean value and standard deviation of the MAE and PSNR measures for the rectification of 25 multiocular image sets captured by different camera pairs, with respect to the reference camera 1. It can be seen that the rectification error produced by Yang’s method slightly decreases as the baseline increases. However, the proposed method improves the performance of Yang’s method in terms of the rectification error by 92.5% for the camera pair with the shortest baseline and by 83.2% for the camera pair with the largest baseline. Additionally, the proposed approach induces significantly less perspective distortion in the rectified images compared to Yang’s method, producing orthogonality angle values $\theta \approx 90^{\circ }$ and aspect ratio values AR≈1.

### 4.2. Multiocular Disparity Estimation Evaluation

The performance of the proposed method for disparity estimation is evaluated in 25 multiocular image sets from the Middlebury dataset [34,35,36]. These test images present challenging conditions for disparity estimation, including low-textured regions, partial occlusions, perspective variations, and depth discontinuities. Each image set consists of five stereo images, with each camera positioned at a 40 mm incremental baseline relative to the first camera, along with the corresponding ground truth disparity maps. The accuracy of disparity estimation is objectively measured using the mean absolute error (MAE) and peak signal-to-noise ratio (PSNR) between the estimated and ground truth disparity maps. The performance of the proposed method is compared with that of the existing method by Li et al. [15].

**Figure 7:**
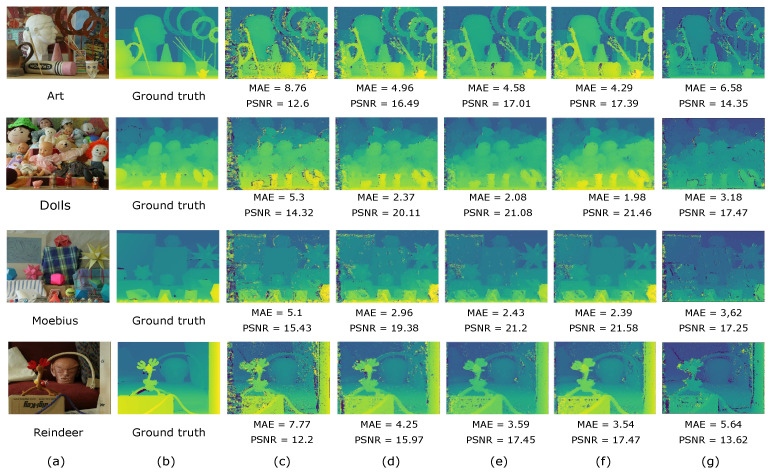
Disparity estimation results for multiocular images obtained with the proposed approach and the method by Li et al. [15]. (**a**) Reference image of the input multiocular image set. (**b**) Ground truth disparity map of the reference image with the largest baseline. Estimated disparity maps obtained with the proposed method for: (**c**) Cameras 1 and 5. (**d**) Cameras 1, 3, and 5. (**e**) Cameras 1, 2, 3, and 5. (**f**) All images. (**g**) Estimated disparity obtained with the method by Li et al. [15].

To estimate the disparity maps using the proposed method, the minimum and maximum disparity values $\tau_{\min}$, $\tau_{\max}$ in Equation (34) are set to those provided by the Middlebury dataset. In addition, a value of α=1.96 is used to set a 95% confidence for the optimized search range in Equation (34). Figure 7a presents the reference images of four multiocular test sets from the Middlebury database. Figure 7b shows the corresponding ground truth disparity maps for the camera pairs with the largest baseline. Figure 7c–g show the estimated disparity maps for different baselines obtained using the proposed approach and the Li’s method, respectively. Note that the proposed approach significantly outperforms Li’s method in terms of both MAE and PSNR measures when estimating the disparity map for the largest baseline, producing fewer errors at object edges, lightly textured areas, and partially occluded regions. Additionally, the performance of the proposed method improves gradually as the baseline increases, confirming that the optimized search range reduces error propagation and matching errors.

Table 4 presents the mean value and standard deviation of the MAE and PSNR measures for disparity estimation in 25 multiocular image sets using both the proposed approach and Li’s method. These measures are computed considering both the non-occluded points and all corresponding points in the multiocular images. For the proposed approach, the objective measures are computed by gradually increasing the baseline until capturing up to five images. In contrast, Li’s method is limited to only three images with different baselines. It can be seen that the proposed method achieves a lower mean MAE value and a higher mean PSNR value compared to Li’s method, specifically for the case of three captured images. It is important to note that both Li’s method and the proposed method significantly outperform the binocular approach. Moreover, the performance of the proposed method improves notably with increasing baseline in terms of both MAE and PSNR measures.

**Figure 8:**
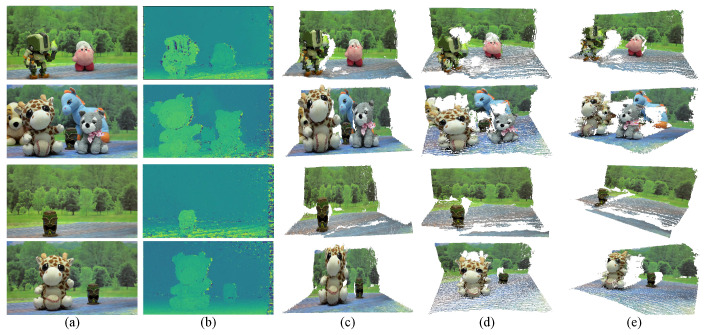
Three-dimensional reconstruction results obtained with the proposed approach in real scenes captured with the experimental platform shown in Figure 5. (**a**) Reference images of the captured scenes. (**b**) Estimated disparity map obtained with the proposed approach between cameras 1 and 4. (**c**–**e**) Different perspective views of the reconstructed three-dimensional scenes.

**Figure 9:**
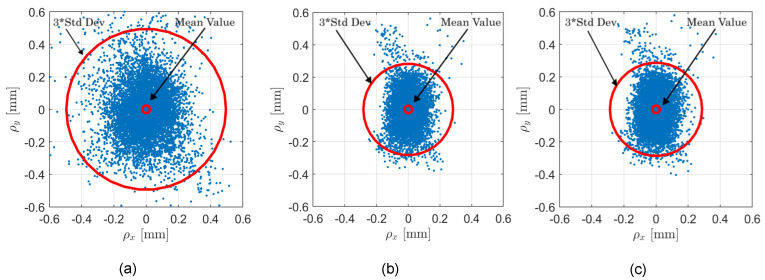
Reprojection errors obtained with the estimated intrinsic parameters obtained using the calibration methods: (**a**) DLT. (**b**) Distorted pinhole. (**c**) Zhang’s method.

The feasibility of the proposed method for multiocular disparity estimation with an adjustable baseline is validated by performing 3D reconstruction of real scenes captured using the experimental platform shown in Figure 5. Figure 8a shows the first of four scene images captured with the multiocular camera, as depicted in Figure 5. These captured images present challenges for rectification and disparity estimation, including perspective variations, lens distortions, varied textures, and depth discontinuities. For each scene, the four captured images were rectified using the method in Section 3.1. Afterwards, the disparity map for the largest baseline was obtained using the multiocular method in Section 3.2. The resultant disparity maps for each scene are shown in Figure 8b. Next, the disparity maps were refined using the algorithm by Aleotti et al. [45]. Finally, the 3D point cloud for each scene was computed from the refined disparity maps using Equations (11) and (12). To quantify the accuracy of the computed 3D scene points, we measured the reprojection error using intrinsic parameter matrices obtained through the Direct Linear Transformation (DLT) [1], the distorted pinhole method [43], and the method by Zhang [26]. The results are presented in Figure 9.

The DLT method produced the highest mean reprojection error of $\mu_{\mathrm{rerr}}=\left(-1.05,3.46\right)\times 10^{-3}$ mm, with a standard deviation of $\sigma_{\mathrm{rerr}}=0.165$ mm, as shown in Figure 9a. The distorted pinhole method yielded a mean reprojection error of $\mu_{\mathrm{rerr}}=\left(0.71,-0.41\right)\times 10^{-3}$ mm and a standard deviation of $\sigma_{\mathrm{rerr}}=0.094$ mm, as shown in Figure 9b. The method by Zhang produced a mean reprojection error of $\mu_{\mathrm{rerr}}=\left(0.49,0.62\right)\times 10^{-3}$ mm and a standard deviation of $\sigma_{\mathrm{rerr}}=0.096$ mm, as shown in Figure 9c. Both the Zhang and distorted pinhole methods produced very similar results. Considering that the nominal error for 3D reconstruction by conventional stereo vision systems is on the order of one millimeter [46], and the obtained reprojection error in the 3D space is 0.1 mm, the differences in calibration accuracy between Zhang’s and the distorted pinhole methods are unperceptible. Additionally, we used the estimated intrinsic camera parameters from the Zhang’s method to compute spatial scene points, owing to its accuracy and accessibility in widely used programming languages such as Python 3.12, MATLAB 24.2, and OpenCV 4.10.0. Figure 8c–e shows different perspective views of the obtained 3D reconstructions using the proposed approach. These results confirm that the proposed method for disparity estimation using multiocular vision with an adjustable baseline is an accurate, robust, and reliable tool for challenging 3D imaging applications.

The flexibility of the proposed method makes it suitable for various applications, including object detection and distance estimation in autonomous navigation, object tracking in surveillance, human–computer interaction, and augmented reality. In medical imaging, it can support multi-angle endoscopy and 3D microscopy, reducing the need for invasive procedures. In industrial settings, it can support quality control in manufacturing, robotic vision for assembly lines, and monitoring systems for infrastructure maintenance and inspection.

### 4.3. Challenges and Considerations

The proposed approach demonstrates strong performance in multiocular image rectification, disparity estimation with baseline adjustment, and spatial point computation. However, there are several challenges and considerations worth discussing.

A multiocular setup of $N_{c}$ cameras aligned horizontally is considered, as illustrated in Figure 1. Additionally, it is assumed that all cameras have the same intrinsic parameters.

The proposed multiocular rectification method depends on detecting corresponding points. Outliers in point correspondences can lead to rectification errors. This issue can be mitigated employing outlier detection algorithms, such as RANSAC [38]. In addition, PSO-based rectification improves robustness by effectively exploring the solution space and avoiding suboptimal solutions. The selection of appropriate PSO configuration parameters impacts both accuracy and convergence speed, enabling a balanced trade-off between them.

The proposed disparity estimation method employs gradual baseline increments. Although many small baseline increments reduce disparity estimation errors by minimizing perspective changes in captured images, they also increase computational cost. Instead, fewer large baseline increments lower the computational cost but increase the risk of disparity estimation errors.

For multiocular imaging, the generic pinhole model is adopted, characterized by focal length, pixel size, principal point, and skewness. Although effective for general purpose cameras, this model does not account for radial or tangential distortions, which can cause misalignment errors of corresponding points, impacting rectification accuracy and increasing disparity estimation errors. These issues can be mitigated by applying a lens distortion correction algorithm during the calibration process or by adopting a more advanced camera model [43].

A key advantage of the proposed multiocular method for disparity estimation is its suitability for implementation on dedicated hardware, such as Graphics Processing Units (GPUs) or Field-Programmable Gate Arrays (FPGAs), exploiting massive parallelism. This enables significant speedup, making it well-suited for real-time applications.

## 5. Conclusions

A reliable method for disparity estimation using multiocular images with an adjustable baseline was presented. First, stereo images with different baselines, captured by a multiocular system, were rectified using a proposed method based on particle swarm optimization. The rectification method minimized an objective function that balanced the rectification error and the induced perspective distortion. Then, an initial dense disparity map was obtained from the pair of rectified images with the shortest baseline. This initial disparity map was then used to estimate the disparity map for rectified images with a larger baseline within a short, optimized search range. This procedure was iterated until the disparity map for the rectified images with the largest baseline was obtained. The proposed search range optimization minimized the propagation of disparity errors and the probability of matching errors.

The performance of the proposed rectification and disparity estimation methods was rigorously evaluated using multiocular images from well-known datasets, as well as images captured on an experimental platform. The proposed rectification method was compared with three existing state-of-the-art methods, demonstrating significant performance improvements in rectification error for binocular images, with a 25% improvement over the method by Fusiello et al. [39], 26.7% over the SPR method [27], and 24% over the DSR method [41]. For multiocular images, the proposed method achieved an 83.2% improvement in rectification error compared to the method by Yang et al. [44]. Additionally, the performance of the proposed approach for disparity estimation in multiocular images with an adjustable baseline was evaluated and compared to a similar existing method. The results indicated that the proposed approach reliably estimated disparity maps from multiocular images with high accuracy, outperforming the method by Li et al. [15] by 19.9% in MAE and 10.1% in PSNR.

Finally, the feasibility of the proposed method for multiocular disparity estimation with an adjustable baseline was demonstrated by performing 3D reconstructions of real scenes captured on the experimental platform. The results confirmed that the proposed approach is suitable for challenging 3D imaging applications. Future work will focus on integrating massive parallel processing for high-rate applications and conduct extensive testing in real-world scenarios.

## Figures and Tables

**Table 1:** Summary of representative techniques in stereo vision.

Focus	Advantages	Disadvantages	References
Depth Perception	Highly accurate for depth estimation of small objects. Robust performance in textureless scene regions. Fast data acquisition.	Requires precise calibration of multiple devices. Dependence on specialized hardware. Susceptible to illumination disturbances.	[3,21,22]
Disparity Estimation	Low computational complexity. Suitable for outdoor applications. Simple calibration. Cost effective.	Matching accuracy is sensitive to the viewing angle. Requires textured images for accurate performance. Large baselines can induce object occlusions.	[1,2]
Multiocular Vision	Less susceptible to object occlusions. High-resolution disparity map. Wide field of view.	Computationally demanding. Susceptible to error propagation in disparity estimation.	[5,8,9,10,11,12]

**Table 2:** Statistical results of the proposed and existing methods for image rectification on forty dataset stereo images in terms of rectification error $e_{rec}$ (in pixels), orthogonality angle θ (in degrees), and Aspect Ratio (AR, unitless).

Rectification Method									
		Fusiello et al. [39]		SPR [27]		DSR [41]		Proposed	
Database	Metric	Mean	St. Dev.	Mean	St. Dev.	Mean	St. Dev.	Mean	St. Dev.
USCML	$e_{rec}$	0.84	0.25	0.86	0.31	0.83	1.06	0.63	0.14
	$\theta (90^{\circ })$	95.4	6.83	87.9	11.9	90.01	0.22	90.05	0.08
	AR (1)	1.2	0.16	1.01	0.05	1.00	0.003	0.99	0.002
Syntim	$e_{rec}$	0.79	1.28	1.15	0.61	0.82	0.8	0.74	0.54
	$\theta (90^{\circ })$	92.7	6.25	86	4.84	89.82	1.29	89.9	0.54
	AR (1)	1.04	0.09	0.96	0.05	0.99	0.02	0.99	0.008

**Table 3:** Statistical results of the proposed and existing methods for image rectification on twenty-five real multiocular images in terms of rectification error $e_{rec}$ (in pixels), orthogonality angle θ (in degrees), and Aspect Ratio (AR, unitless).

Multiocular Rectification							
		Cameras 1–2		Cameras 1–3		Cameras 1–4	
Method	Metric	Mean	St. Dev.	Mean	St. Dev.	Mean	St. Dev.
Yang et al. [44]	$e_{rec}$	4.45	0.08	4.22	0.15	3.10	0.20
	$\theta (90^{\circ })$	95.68	0.03	95.64	0.07	95.62	0.08
	AR (1)	1.01	0.005	1.09	0.001	1.01	0.001
Proposed	$e_{rec}$	0.33	0.40	0.42	0.23	0.52	0.50
	$\theta (90^{\circ })$	90.01	0.03	89.99	0.01	90.01	0.05
	AR (1)	1.01	0.01	0.99	0.02	1.02	0.01

**Table 4:** Statistical results of the proposed approach and the method by Li et al. [15] for disparity estimation in twenty-five multiocular dataset images.

		Non-Occluded Points				All Points			
		MAE		PSNR		MAE		PSNR	
Method	Number of Images	Mean	Std. Dev.	Mean	Std. Dev.	Mean	Std. Dev.	Mean	Std. Dev.
Proposed	2	2.64	1.80	17.67	3.96	5.60	2.22	13.90	1.85
	3	1.53	1.22	23.13	4.87	3.16	1.64	17.87	2.55
	4	1.47	1.16	23.64	5.21	2.76	1.72	19.55	3.38
	5	1.44	1.19	23.98	5.04	2.59	1.80	19.75	3.2
Li et al. [15]	3	1.91	1.47	20.66	4.04	3.78	1.97	15.81	2.27

## Data Availability

Publicly available datasets were analyzed in this study. Middlebury stereo dataset: https://vision.middlebury.edu/stereo/ (accessed on 16 May 2024). INRIA Syntim dataset: http://perso.lcpc.fr/tarel.jean-philippe/syntim/paires.html (accessed on 3 May 2024). MCL-RS dataset: https://mcl.usc.edu/mcl-rs-database/ (accessed on 7 May 2024).

## Abbreviations

The following abbreviations are used in this manuscript:

3D	Three-Dimensional
SAD-Census	Sum of Absolute Difference Census
AMC	Adaptive Morphological Correlation
PSO	Particle Swarm Optimization
eval	Evaluation
diss	Dissimilarity
BDMR	Binary-Dissimilarity-to-Matching Ratio
MCL-RS	Media Communication Lab Real Stereo
SIFT	Scale-Invariant Feature Transform
AR	Aspect Ratio
DSR	Direct Self-Rectification
SPR	Stereo Phase Rectification
MAE	Mean Value
DLT	Direct Linear Transform
MAE	Mean Absolute Error
PSNR	Peak Signal-to-Noise Ratio
St. Dev.	Standard Deviation

## Appendix A

### Particle Swarm Optimization

Particle swarm optimization (PSO) is a population-based technique that is highly effective in solving diverse real-world optimization problems. In PSO, an ensemble of *N* potential solutions, called particles, is initialized by randomly sampling the solution space $\Omega_{v}$. This ensemble, known as the swarm, undergoes to iterative refinement by applying search operators until it converges to an optimal solution that meets predefined termination criteria. Let $V=\{v_{i}|i=1,\ldots ,N_{v}\}$ be the particle swarm, where each particle is represented by a position vector $v_{i}={\left[v_{1,i},v_{2,i},\ldots ,v_{d_{1},i}\right]}^{T}$ defined within $\Omega_{v}$. The proximity of a particle to the optimal solution is quantified by a fitness function *J*, which maps $J\left(v_{i}\right):R^{d_{1}}\to R$. When applying PSO to solve an application problem, each particle $v_{i}$ encodes a solution. Therefore, a clear definition of $\Omega_{v}$ is crucial. Let $\Omega_{G}$ be a $d_{2}$-dimensional space where all potential solutions $G_{i}$ to the application problem are defined. In this manner, for every problem solution $G_{i}\in \Omega_{G}$, there exists a particle $v_{i}\in \Omega_{v}$ such that $T\left(v_{i}\right)=G_{i}$, where T performs the mapping $T\left(v_{i}\right):R^{d_{1}}\to R^{d_{2}}$.

The search operators are essential in PSO. These operators drive the particles through the solution space during each iteration. The search operators serve a dual purpose: promoting diversification in global search and intensifying local search when promising solutions are identified. By evaluating the particles using a fitness function *J*, the search operators update their positions in the next iteration, moving them closer to the global solution. In PSO, the positions $v_{i}^{k}$ and velocities $\tau_{i}^{k}$ of a particle $v_{i}$ are updated from the current iteration *k* to the next iteration k+1 as [47]

(A1) $$\begin{array}{cc} v_{i}^{k+1} & =v_{i}^{k}+\tau_{i}^{k+1},\\ \tau_{i}^{k+1} & =\omega \tau_{i}^{k}+\alpha_{1}c_{1}\left(v_{b,i}^{k}-v_{i}^{k}\right)+\alpha_{2}c_{2}\left(v_{g,i}^{k}-v_{i}^{k}\right), \end{array}$$

where $v_{b,i}$ is the best-evaluated particle so far, $v_{g,i}$ is the best-evaluated particle of the swarm in the *k*-th iteration, ω is an inertia coefficient, $c_{1}$ and $c_{2}$ are the cognitive and social acceleration coefficients, respectively. Additionally, $\alpha_{1}$ and $\alpha_{2}$ are random variables uniformly distributed over the interval [0,1]. Note that the position of a particle in $\Omega_{v}$ determines its fitness value $J(v_{i})$, and the current position and velocity of the particles influence their new position in the subsequent iteration.

## References

[1] Hartley R., Zisserman A. (2003). Multiple View Geometry in Computer Vision.

[2] Scharstein D., Szeliski R. (2002). A taxonomy and evaluation of dense two-frame stereo correspondence algorithms. Int. J. Comput. Vision.

[3] Luo H., Pape C., Reithmeier E. (2020). Scale-Aware Multi-View Reconstruction Using an Active Triple-Camera System. Sensors.

[4] Hirschmuller H. (2008). Stereo Processing by Semiglobal Matching and Mutual Information. IEEE Trans. Pattern Anal. Mach. Intell..

[5] Wang J., Peng C., Li M., Li Y., Du S. (2022). The study of stereo matching optimization based on multi-baseline trinocular model. Multimed. Tools Appl..

[6] Banks J., Corke P. (2001). Quantitative evaluation of matching methods and validity measures for stereo vision. Int. J. Robot. Res..

[7] Fife W.S., Archibald J.K. (2013). Improved census transforms for resource-optimized stereo vision. IEEE Trans. Circuits Syst. Video Technol..

[8] Okutomi M., Kanade T. (1993). A multiple-baseline stereo. IEEE Trans. Pattern Anal. Mach. Intell..

[9] Sakamoto S., Cox I.J., Tajima J. (1997). A multiple-baseline stereo for precise human face acquisition. Pattern Recognit. Lett..

[10] Bunschoten R., Krose B. (2003). Robust scene reconstruction from an omnidirectional vision system. IEEE Trans. Robot. Autom..

[11] Liu F., Zhou S., Wang Y., Hou G., Sun Z., Tan T. (2020). Binocular Light-Field: Imaging Theory and Occlusion-Robust Depth Perception Application. IEEE Trans. Image Process..

[12] Rathnayaka P., Park S.Y. (2020). iGG-MBS: Iterative Guided-Gaussian Multi-Baseline Stereo Matching. IEEE Access.

[13] Hou Y., Liu C., An B., Liu Y. (2022). Stereo matching algorithm based on improved census transform and texture filtering. Optik.

[14] Wang Y., Gu M., Zhu Y., Chen G., Xu Z., Guo Y. (2022). Improvement of AD-Census Algorithm Based on Stereo Vision. Sensors.

[15] Li J., Li Z., Zhao H. (2021). Efficient global stereo-matching method of general images under a long baseline based on baseline estimation. Appl. Opt..

[16] Poggi M., Tosi F., Batsos K., Mordohai P., Mattoccia S. (2021). On the synergies between machine learning and binocular stereo for depth estimation from images: A survey. IEEE Trans. Pattern Anal. Mach. Intell..

[17] Cheng C., Li H., Zhang L. (2021). Two-branch convolutional sparse representation for stereo matching. IEEE Access.

[18] Qu Z., Li L., Jin W., Yang Y. (2024). Real-time binocular visual localization system based on the improved BGNet stereo matching framework. JOSA A.

[19] Zhang Y., Zheng Y., Ling Y., Meng H., Chen G. (2023). A robust and real-time DNN-based multi-baseline stereo accelerator in FPGAs. J. Syst. Architect..

[20] Wu R., Wang M., Li Z., Zhou J., Chen F., Wang X., Sun C. (2024). Few-Shot Stereo Matching with High Domain Adaptability Based on Adaptive Recursive Network. Int. J. Comput. Vision.

[21] Fu T., Yu H., Yang W., Hu Y., Scherer S. (2022). Targetless extrinsic calibration of stereo, thermal, and laser sensors in structured environments. IEEE Trans. Instrum. Meas..

[22] Zhang J., Han F., Han D., Yang J., Zhao W., Li H. (2024). Integration of Sonar and Visual–Inertial Systems for SLAM in Underwater Environments. IEEE Sens. J..

[23] Mezura-Montes E., Coello Coello C.A. (2011). Constraint-handling in nature-inspired numerical optimization: Past, present and future. Swarm Evol. Comput..

[24] Diaz-Ramirez V.H., Gonzalez-Ruiz M., Kober V., Juarez-Salazar R. (2022). Stereo image matching using adaptive morphological correlation. Sensors.

[25] Juarez-Salazar R., Diaz-Ramirez V.H. (2017). Operator-based homogeneous coordinates: Application in camera document scanning. Opt. Eng..

[26] Zhang Z. (2000). A flexible new technique for camera calibration. IEEE Trans. Pattern Anal. Mach. Intell..

[27] Juarez-Salazar R., Rios-Orellana O.I., Diaz-Ramirez V.H. (2022). Stereo-phase rectification for metric profilometry with two calibrated cameras and one uncalibrated projector. Appl. Opt..

[28] Lu J., Cai H., Lou J.G., Li J. (2007). An Epipolar Geometry-Based Fast Disparity Estimation Algorithm for Multiview Image and Video Coding. IEEE Trans. Circuits Syst. Video Technol..

[29] Cyganek B., Siebert J. (2011). An Introduction to 3D Computer Vision Techniques and Algorithms.

[30] Hamzah R.A., Ibrahim H. (2016). Literature Survey on Stereo Vision Disparity Map Algorithms. J. Sens..

[31] Gómez V., Maravall A. (1994). Estimation, Prediction, and Interpolation for Nonstationary Series with the Kalman Filter. J. Am. Stat. Assoc..

[32] Ko H., Shim H.S., Choi O., Kuo C.C.J. (2017). Robust uncalibrated stereo rectification with constrained geometric distortions (USR-CGD). Image Vis. Comput..

[33] Isgro F., Trucco E. Projective rectification without epipolar geometry. Proceedings of the 1999 IEEE Computer Society Conference on Computer Vision and Pattern Recognition.

[34] Scharstein D., Pal C. (2007). Learning conditional random fields for stereo. Proceedings of the 2007 IEEE Conference on Computer Vision and Pattern Recognition.

[35] Hirschmuller H., Scharstein D. (2007). Evaluation of cost functions for stereo matching. Proceedings of the 2007 IEEE Conference on Computer Vision and Pattern Recognition.

[36] Scharstein D., Hirschmüller H., Kitajima Y., Krathwohl G., Nešić N., Wang X., Westling P. (2014). High-resolution stereo datasets with subpixel-accurate ground truth. Proceedings of the German conference on pattern recognition.

[37] Lowe G. (2004). Distinctive Image Features from Scale-Invariant Keypoint. Int. J. Comput. Vis..

[38] Sangappa H.K., Ramakrishnan K.R. (2019). A Probabilistic Analysis of a Common RANSAC Heuristic. Mach. Vis. Appl..

[39] Fusiello A., Irsara L. (2011). Quasi-Euclidean epipolar rectification of uncalibrated images. Mach. Vis. Appl..

[40] Mallon J., Whelan P.F. (2005). Projective rectification from the fundamental matrix. Image Vis. Comput..

[41] Xiao R., Sun W., Pang J., Yan Q., Ren J. DSR: Direct Self-Rectification for Uncalibrated Dual-Lens Cameras. Proceedings of the 2018 International Conference on 3D Vision (3DV).

[42] Fusiello A., Roberto V., Trucco E. (2001). Symmetric stereo with multiple windowing. Int. J. Pattern Recogn..

[43] Juarez-Salazar R., Zheng J., Diaz-Ramirez V.H. (2020). Distorted pinhole camera modeling and calibration. Appl. Opt..

[44] Yang J., Ding Z., Guo F., Wang H. (2014). Multiview image rectification algorithm for parallel camera arrays. J. Electron. Imaging.

[45] Aleotti F., Tosi F., Zama Ramirez P., Poggi M., Salti S., Di Stefano L., Mattoccia S. Neural Disparity Refinement for Arbitrary Resolution Stereo. Proceedings of the International Conference on 3D Vision.

[46] Wang T.M., Shih Z.C. (2021). Measurement and analysis of depth resolution using active stereo cameras. IEEE Sens. J..

[47] Poli R., Kennedy J., Blackwell T. (2007). Particle swarm optimization. Swarm Intell..

